# Individual risk of cutaneous melanoma in New Zealand: developing a clinical prediction aid

**DOI:** 10.1186/1471-2407-14-359

**Published:** 2014-05-22

**Authors:** Mary Jane Sneyd, Claire Cameron, Brian Cox

**Affiliations:** 1 Hugh Adam Cancer Epidemiology Unit, Department of Preventive and Social Medicine, Dunedin School of Medicine, University of Otago, P.O. Box 56, Dunedin 9054, New Zealand; 2 Department of Preventive and Social Medicine, Dunedin School of Medicine, University of Otago, P.O. Box 913, Dunedin 9054, New Zealand

**Keywords:** Melanoma, Absolute risk, Risk prediction, Logistic model, Risk assessment

## Abstract

**Background:**

New Zealand and Australia have the highest melanoma incidence rates worldwide. In New Zealand, both the incidence and thickness have been increasing. Clinical decisions require accurate risk prediction but a simple list of genetic, phenotypic and behavioural risk factors is inadequate to estimate individual risk as the risk factors for melanoma have complex interactions. In order to offer tailored clinical management strategies, we developed a New Zealand prediction model to estimate individual 5-year absolute risk of melanoma.

**Methods:**

A population-based case–control study (368 cases and 270 controls) of melanoma risk factors provided estimates of relative risks for fair-skinned New Zealanders aged 20–79 years. Model selection techniques and multivariate logistic regression were used to determine the important predictors. The relative risks for predictors were combined with baseline melanoma incidence rates and non-melanoma mortality rates to calculate individual probabilities of developing melanoma within 5 years.

**Results:**

For women, the best model included skin colour, number of moles > =5 mm on the right arm, having a 1st degree relative with large moles, and a personal history of non-melanoma skin cancer (NMSC). The model correctly classified 68% of participants; the C-statistic was 0.74. For men, the best model included age, place of occupation up to age 18 years, number of moles > =5 mm on the right arm, birthplace, and a history of NMSC. The model correctly classified 67% of cases; the C-statistic was 0.71.

**Conclusions:**

We have developed the first New Zealand risk prediction model that calculates individual absolute 5-year risk of melanoma. This model will aid physicians to identify individuals at high risk, allowing them to individually target surveillance and other management strategies, and thereby reduce the high melanoma burden in New Zealand.

## Background

New Zealand and Australia are the two countries with the highest melanoma incidence rates in the world. In 2009, New Zealand had an age-standardised rate (ASR) of 42.8 per 100,000 in men and 33.6 per 100,000 in women [1]. Despite over 20 years of health promotion campaigns predominantly focusing on melanoma prevention, and ad hoc opportunistic screening by skin examination, both the rates [2] and the thickness of melanomas have been increasing in Maori and non-Maori New Zealanders [3]. Notwithstanding these worrying trends, as thickness is the major prognostic factor, the best avenue for reducing melanoma mortality remains early diagnosis while the lesion is still thin [4].

It is generally believed that screening of high-risk people by total skin examination for early detection is more feasible, cheaper, has fewer false positive screens and lower patient anxiety [5] compared to population screening. However, screening of high-risk people requires their accurate identification.

Risk assessment and prognostication are regularly used in medicine to guide management decisions. Nevertheless, accurately predicting disease development is challenging, and the common practice of stratifying individual risk based on a single variable, such as age, rarely gives a precise enough estimate of individual risk. Although many risk factors for melanoma are well described, their multiple interactions make risk prediction complex. However, having estimated an individual’s absolute risk by consideration of their personal combination of risk factors, appropriate strategies for prevention, surveillance and early diagnosis can be offered. By encouraging joint patient-clinician decisions on these strategies it is hoped to improve melanoma control, particularly in people at high and very high risk, and thus reduce morbidity and mortality from melanoma in New Zealand.

As an aid to clinical decision-making, we have developed a personal risk assessment model that estimates the probability of an individual developing their first melanoma within the next 5 years. The model is based on results from a New Zealand population-based case–control study of risk factors for melanoma.

## Methods

The data from a 1992–1994 case–control study of skin screening and melanoma risk factors in three geographic regions of New Zealand [6] provided the relative risk estimates for melanoma.

### Case–control study

Histology reports of melanomas were obtained directly from pathology laboratories in the Bay of Plenty (population 117,500; lat 38°S, long 177°E), Hawkes Bay (population 111,000; lat 39°S, long 177°E) and Nelson-Marlborough (population 104,000; lat 41.5°S, long 174°E) regions of New Zealand from 1 July 1992 to 30 June 1994. In addition, research nurses manually searched laboratory files for missed reports. Finally, all melanoma registrations of the New Zealand Cancer Registry that were not identified through the laboratories, were also included. Cases aged from 20 to 79 years, with a first diagnosis of in-situ, invasive or metastatic cutaneous melanoma, were interviewed within one year of diagnosis. Eligible interviews were completed for 368 cases.

Control subjects were randomly selected from the electoral roll. In New Zealand all residents aged 18 years or older have to be enrolled on an electoral roll by law. The rolls are about 95% complete for residents aged 30 years and over. Controls were frequency matched by age and region to cases accrued in the first 6 months of the study, and those with a history of melanoma were excluded. Eligible interviews were completed for 270 controls.

Trained interviewers, using a standardised telephone interview, collected data on demographics, melanoma risk factors, previous medical history, family history of skin cancer, experience of screening skin examinations, knowledge of melanoma, and for cases, diagnostic histories. Participants self-assessed their freckling and mole numbers prior to interview, according to a mailed protocol. No clinical examinations were performed. A week before interview, participants were mailed an information pack including photographs of moles to help with identification, a transparent plastic measurement card to measure their moles, and diagrams of body parts with different densities of moles and freckles. In a separate study we compared our method of self-assessment with clinical examination and found very good agreement for large moles (Kappa 0.83).

Ethnicity and phenotype data for all subjects were collected by self-report during the interview; participants with coloured or dark phenotype were excluded.

The protocol was approved by the three regional ethics committees (the Bay of Plenty Regional Ethics Committee, the Hawkes Bay Regional Ethics Committee, and the Nelson/Marlborough Regional Ethics Committee). All participants gave informed consent to take part in the case–control study.

### Variables in the logistic model

From the original variables in the case–control study we selected 44 of interest based on published risk factors for melanoma, previous analysis of the case–control study, and ease of collection in primary care, including several measures of sun exposure, skin reaction to sun and phenotype (see Additional file 1). Chi-square tests and univariate logistic regression were used to measure the association between each categorical variable and case–control status. Pearson correlation coefficients measured correlations between continuous variables and chi-square tests between categorial variables. From the 44 variables of interest, 9 candidate variables for women and 8 candidate variables for men (Table 1) had p-values <0.25 in univariate tests of association so were considered for inclusion in the final multivariate models [7]. The mole count on the right arm was included as a continuous variable in the model for men. However, as one woman reported a very high but unspecified number of moles on her right arm, this variable was treated as a categorical variable for women*.* Some variables of interest were excluded as they occurred infrequently (for example, having a 1st degree relative with a history of melanoma), or they were highly correlated with or were linear functions of another variable (for example, the number of large moles on the body and the number of large moles on the right arm). Very few data were missing. Cases and controls with missing data were excluded unless otherwise specified: no missing data were imputed.

**Table 1:** Candidate variables and associated chi-square statistics and p-values, for men and women

		**Women**				**Men**			
		**Controls n (%)**	**Cases n (%)**	**Chi-square statistic**	**p**	**Controls n (%)**	**Cases n (%)**	**Chi-square statistic**	**p**
Age at diagnosis (AGE)	<=50 years	57 (41)	93 (51)	3.22	0.073	69 (53)	53 (28)	18.98	<0.001
	> 50 years	82 (59)	89 (49)			62 (47)	133 (72)		
Had blistering sunburn (SUNBURN)		71 (51)	118 (65)	6.16	0.013				
Teenage hair colour (HAIRCOLOUR)	Black/brown	89 (64)	89 (49)	7.30	0.007				
	Fair/blond/red	50 (36)	93 (51)						
Eye colour (EYECOLOUR)	Brown	25 (18)	20 (11)	5.26	0.154	17 (13)	39 (21)	5.20	0.158
	Hazel	30 (22)	31 (17)			29 (22)	30 (16)		
	Green	20 (14)	29 (16)			17 (13)	30 (16)		
	Grey/blue	64 (46)	102 (56)			68 (52)	87 (47)		
Skin colour (SKINCOLOUR)	Olive	22 (16)	7 (4)	18.50	<0.001	12 (9)	17 (9)	3.88	0.144
	Medium	53 (38)	57 (31)			56 (43)	60 (32)		
	Fair	64 (46)	118 (65)			63 (48)	109 (59)		
Freckling (FRECKLING)	None	77 (55)	73 (40)	14.19	0.003				
	Few	45 (32)	60 (33)						
	Moderate	10 (7)	18 (10)						
	Many	7 (5)	31 (17)						
Number moles > =5 mm on right arm (MOLES_RARM)	0	96 (69)	88 (48)	21.76	<0.001	Continuous variable		1.15 (odds ratio)	0.048
	1	23 (17)	30 (16)						
	2	11 (8)	22 (12)						
	3+	8 (6)	41 (23)						
1st degree relative with large or unusual moles (FAMHXMOLES)		56 (40)	116 (64)	17.44	<0.001				
Personal history of NMSC (NMSC)		5 (4)	20 (11)	6.00	0.014	5 (4)	28 (15)	10.41	0.001
Occupation < =18 years (OCC < =18)	Indoor					44 (34)	43 (23)	7.14	0.028
	Indoor and outdoor					52 (40)	67 (36)		
	Outdoor					35 (27)	74 (40)		
Occupation >18 years (OCC > 18)	Indoor					44 (34)	43 (23)	6.97	0.031
	Indoor and outdoor					48 (37)	61 (33)		
	Outdoor					39 (30)	80 (43)		
Birthplace (BIRTHPLACE)	Outside NZ					23 (18)	15 (8)	6.57	0.010
	In NZ					108 (82)	171 (92)		

### Statistical methods

All analyses were performed in STATA: release 11 [8]. The analytic method of Fears et al. was used [9].

There were 3 components to the calculation of the 5-year absolute risk:

1. The development of logistic models, separately for men and women, to provide estimates of relative risk (RR), from odds ratios, for risk factors identified in the case–control study.

2. The calculation of the attributable risk (AR), using data from the cases only and the RRs from the logistic model, using the method of Bruzzi et al. [10].

3. The use of an estimating equation (described by Gail et al. [11]) incorporating baseline melanoma incidence and non-melanoma mortality to provide individualised estimates of 5-year absolute risk of melanoma.

To avoid unstable models and unreliable assessment of model performance [12] instead of building the logistic models from a subset of the dataset and validating the model on the remainder, the models were developed from the entire dataset. As these models were developed for use in primary care, smaller models with fewer variables to measure were preferred for practical reasons. Model selection techniques described below were used to determine the best and most parsimonious logistic model from the variables and 1st order interaction terms. We used backward selection with the likelihood ratio statistic [7] as the exclusion criterion for nested models, and Akaike’s Information Criterion (AIC) as the exclusion criterion for non-nested models. For internal validation, forward selection was also used, and the validity of variable selection was assessed by backward and forward bootstrap resampling [13]. Calibration was examined by plotting observed versus expected probabilities for deciles of individual predicted risk [14] and assessed by the Hosmer-Lemeshow test [7]; the linktest [8] was used to test the specification of the model; and a classification test [8] was used to examine sensitivity, specificity and percentage of participants correctly classified. Discrimination was assessed by the C-statistic (the area under the curve of the receiver operating characteristic (ROC)) [15].

From the RRs provided by the logistic models, we calculated the AR and obtained standard errors through bootstrapping (see Additional file 2: Appendix 1).

For estimates of risk by latitude, district health board regions of residence were collated into four groups at different latitudes: North (Northland, Waitemata, Auckland, Counties Manukau), Midland (Waikato, Lakes, Bay of Plenty, Tairawiti, Hawke’s Bay, Taranaki, Whanganui), Central (Mid Central, Capital and Coast, Hutt, Wairarapa, Nelson, Marlborough), and South (West Coast, Canterbury, South Canterbury, Otago, Southland). The regional baseline age- and sex-specific hazard rate of melanoma for people with no risk factors was calculated as the age-, sex- and region-specific incidence (obtained from New Zealand Ministry of Health publications [16]) times one minus the AR.

To include competing mortality (which reduces the absolute risk of developing melanoma) in the calculation of absolute risk, age- and sex-specific non-melanoma mortality rates for 1996–2006 were calculated from publications of the Ministry of Health [16,17]. The baseline incidence rates for people without melanoma risk factors, and non-melanoma mortality rates are shown in Table 2. Using the above information, individual 5-year absolute risks were calculated (see Additional file 2: Appendices 2 and 3).

**Table 2:** Estimated average annual rates per 100,000 person-years for melanoma incidence and non-melanoma mortality for the years 1996–2006 by age group, sex and region of residence

	**WOMEN**					**MEN**				
	**Melanoma incidence**				**Non-melanoma mortality**	**Melanoma incidence**				**Non-melanoma mortality**
**Age group (years)**	**North**	**Midland**	**Central**	**South**	**All of New Zealand**	**North**	**Midland**	**Central**	**South**	**All of New Zealand**
20-24	13.05	22.07	8.57	14.18	37.68	7.33	11.64	5.72	9.79	112.10
25-29	21.14	30.45	15.23	25.44	38.94	16.22	20.84	9.42	12.70	114.83
30-34	27.38	44.99	26.69	36.10	51.39	17.72	26.78	16.59	23.40	103.14
35-39	32.77	44.31	32.03	41.24	64.68	29.41	34.80	23.81	23.30	125.37
40-44	53.17	61.07	48.73	**44.53**	**101.50**	37.77	49.91	34.04	39.90	158.18
45-49	61.20	82.49	70.47	67.58	165.57	55.79	75.69	49.06	54.12	232.20
50-54	75.19	91.40	71.11	65.09	266.10	88.66	95.82	70.32	60.31	372.46
55-59	89.69	106.81	80.01	73.01	429.77	127.45	116.35	89.78	82.20	637.30
60-64	79.87	117.15	85.87	73.78	675.84	150.31	**161.28**	102.88	108.70	**1074.51**
65-69	117.82	130.65	101.78	95.80	1101.00	184.56	197.11	138.76	120.67	1843.10
70-74	124.84	152.85	117.24	97.26	1872.51	223.62	239.07	166.16	171.58	3180.72
75-79	141.09	161.42	122.37	107.45	3248.98	260.94	281.62	214.05	190.06	5288.09
80-84	163.27	174.31	166.46	112.90	5929.85	307.99	308.91	254.41	236.31	8937.20
85+	154.04	176.49	139.02	140.24	14712.65	323.24	337.96	239.09	240.37	17857.80

The figures in bold are used in Additional file 2: Appendix 3.

The data for the current analysis included 368 cases (182 women, 186 men) and 270 controls (139 women, 131 men).

## Results

In the case–control study we received notifications for 604 cutaneous melanoma patients of whom 166 were ineligible to participate (73 were outside the age range, 54 had a previous diagnosis of melanoma, 19 had died, 5 lived outside New Zealand and 15 had mental impairment or deafness preventing them from being interviewed). Of the 438 eligible cases, interviews were completed with 368 giving a response rate of 84.0% and a cooperation rate (number of completed interviews in those whom we could contact) of 84.9% (368/433). Of the 368 cases, 158 had in-situ melanoma at diagnosis and the remainder were invasive. The depth of the invasive lesions ranged from 0.1 mm to 6.1 mm, and 62% of the invasive lesions were < =1 mm at diagnosis.

We approached 573 potential controls of whom 73 were ineligible (32 had dark or coloured skin, 14 lived outside the region, 5 had a previous diagnosis of melanoma, 5 had died, and 17 could not complete a telephone interview because of deafness, history of stroke or other mental impairment). Of the 500 eligible controls, interviews were completed for 270 giving a response rate of 54.0% and a cooperation rate of 67.3% (270/401).

### Logistic model for women

From the results of the case–control analysis, nine candidate variables were considered for the logistic model for women: age group at diagnosis (AGE: <=50/>50 years), ever having had a blistering sunburn (SUNBURN: yes/no), teenage hair colour (HAIRCOLOUR: black, brown/fair, blond, red), eye colour (EYECOLOUR: brown/hazel/green/grey, blue), skin colour (SKINCOLOUR: olive/medium/fair), freckling (FRECKLING: none/few/moderate/many), number of moles > =5 mm on right arm (MOLES_RARM: 0/1/2/3+), 1st degree relative with large moles (FAMHXMOLES: yes/no) and personal history of non-melanoma skin cancer (NMSC: yes/no) (Table 1). The data for these variables were almost complete: only two participants were missing mole counts. After the formal model selection, four variables were independently associated with melanoma risk: SKINCOLOUR, MOLES_RARM, FAMHXMOLES, and NMSC (Table 3). There were no significant first-order interactions between any of these terms. Having fair skin (compared to olive skin) gave the greatest risk of melanoma (Table 3) with an adjusted odds ratio (OR) of 4.50, closely followed by having three or more large moles (versus none) on the right arm (adjusted OR = 4.28), and a personal history of NMSC (adjusted OR = 3.75).

**Table 3:** The variables included in the final logistic models, and associated odds ratios

		**Women**		**Men**	
	**Variable**	**Adjusted relative risk**	**95% CI**	**Adjusted relative risk**	**95% CI**
Skin colour (SKINCOLOUR)					
	Olive	1			
	Medium	2.94	1.11 to 7.79		
	Fair	4.50	1.74 to 11.64		
1st degree relative with large or unusual moles (FAMHXMOLES)					
	Don't know	1			
	No	1.09	0.52 to 2.28		
	Yes	2.62	1.31 to 5.26		
Number moles > =5 mm on right arm (MOLES_RARM)				(Continuous variable for men)	
	0	1		1.15	1.01 to 1.32
	1	1.34	0.69 to 2.61		
	2	2.59	1.15 to 5.86		
	3+	4.28	1.85 to 9.89		
Personal history of NMSC (NMSC)					
	No	1		1	
	Yes	3.75	1.27 to 11.08	3.10	1.13 to 8.50
Age group at diagnosis (AGE)					
	<=50 years			1	
	>50 years			2.59	1.57 to 4.27
Occupation < = 18 years (OCC < =18)					
	Indoor			1	
	Indoor and outdoor			1.55	0.85 to 2.81
	Outdoor			1.94	1.04 to 3.62
Birthplace (BIRTHPLACE)	Outside NZ			1	
	In NZ			2.21	1.05 to 4.66

**Figure 1:**
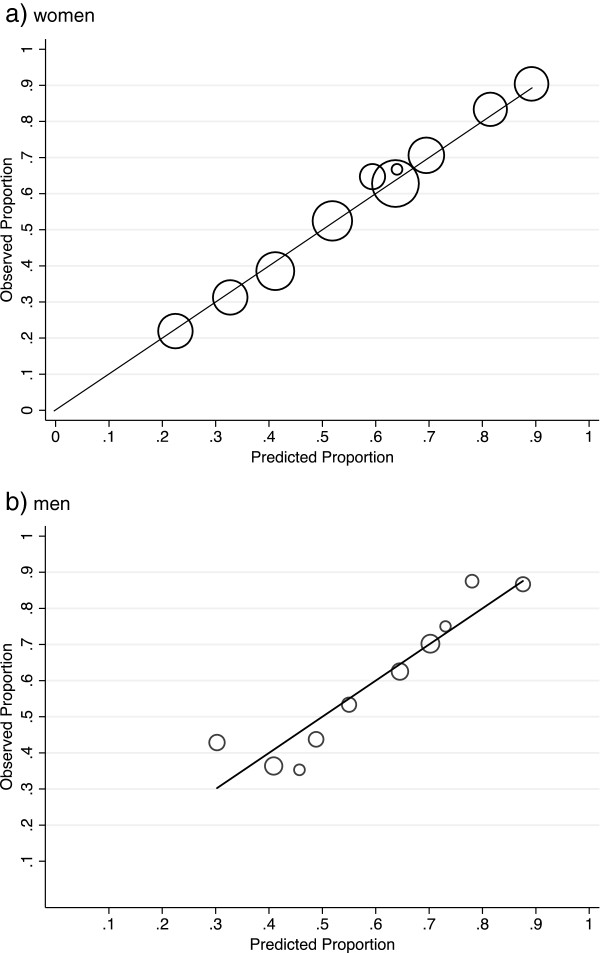
**Observed proportion of melanoma cases compared with the predicted proportion, based on the risk predictor model, for men and women.** Circle size is proportional to the total number of cases in each of 10 data subsets. **a)** women (p for Hosmer-Lemeshow test = 0.99) **b)** men (p for Hosmer-Lemeshow test = 0.70).

The reproducibility of the model assessed by bootstrap re-sampling was very good: about 98% of the models chosen included FAMHXMOLES, 71% and 70% included NMSC and SKINCOLOUR, respectively, and 61% included MOLES_RARM. The diagnostic tests confirmed that the model was appropriately specified and fitted adequately. The model correctly classified 68.3% of participants; discrimination, as shown by the C-statistic, was 0.74; the model exhibited good calibration over the range of predicted probabilities (Figure 1a); and the AR for these variables in women was 0.89 (95% CI: 0.68 to 0.97).

### Logistic model for men

From the case–control study the eight candidate variables considered for the model for men were age group at diagnosis (AGE: <=50/>50 years), outdoor occupation aged < = 18 years (OCC < =18: indoor/indoor and outdoor/outdoor), outdoor occupation after 18 years old (OCC > 18: indoor/indoor and outdoor/outdoor), eye colour (EYECOLOUR: brown/hazel/green/grey, blue), skin colour (SKINCOLOUR: olive/medium/fair), number of moles > =5 mm on the right arm (MOLES_RARM: continuous variable), birthplace (BIRTHPLACE: outside New Zealand/in New Zealand) and personal history of NMSC (NMSC: yes/no) (Table 1). Only 0.6% of data was missing for OCC < =18 and OCC > 18 and none for the other variables. The five variables selected for the final model were AGE, OCC < =18, MOLES_RARM, BIRTHPLACE and NMSC (Table 3). Statistical criteria did not support the inclusion of any interactions.

In men the highest odds of developing melanoma was from a personal history of NMSC (OR = 3.1) whereas AGE > 50 yrs (compared to < =50 years) gave an OR of 2.59 (Table 3).

Bootstrap resampling showed that the model was reproducible. About 96% of fitted models included AGE; 71% of models included BIRTHPLACE; and 72%, 71% and 52% identified MOLES_RARM, NMSC and OCC < =18 as important predictors, respectively. Statistical tests indicated that the model had an adequate fit. There was no evidence of nonlinearity in risk from the number of large moles on the right arm, so this was included as a continuous variable. The model correctly classified 66.7% of participants; the C-statistic was 0.71; the model had good calibration (Figure 1b); and the AR for men was estimated to be 0.85 (95% CI: 0.67 to 0.94).

### 5-year absolute risk calculations

For each person, their RR was estimated from their profile of risk factors. The appropriate baseline incidence and non-melanoma mortality rates were selected from Table 2 based on their age, sex and region of residence, and these were combined (see equation in Additional file 2: Appendix 2) to calculate personal absolute 5-year risk of melanoma. Examples of absolute risks by age and region of residence are shown in Table 4.

**Table 4:** Absolute 5-year risk (%) of developing melanoma by age at diagnosis and region of residence, for various risk profiles

	**Women**				**Men**			
	**20 yrs**	**40 yrs**	**60 yrs**	**80 yrs**	**20 yrs**	**40 yrs**	**60 yrs**	**80 yrs**
	High Risk: RR = 189.38 (fair skin, 3 or more large moles, close family with large moles, personal history of NMSC)				High Risk: RR = 27.19 (age < =50, outdoor occupation < =18 yrs, 5 moles > =5 mm on right arm, born in NZ, history of NMSC); RR = 70.31 (age > 50, outdoor occupation < =18 yrs, 5 moles > =5 mm on right arm, born in NZ, history of NMSC)			
North	1.36	5.41	7.9	13.67	0.15	0.76	7.42	12.15
Midland	2.28	6.19	11.37	14.52	0.24	1.01	**7.94**	12.19
Central	0.89	4.97	8.47	13.92	0.17	0.69	5.14	10.17
South	1.47	4.55	7.32	9.69	0.20	0.81	5.42	9.49
	Medium Risk: RR = 12.66 (fair skin, 2 large moles, no close family with large moles, no personal history of NMSC)				Medium Risk: RR = 12.25 (age < =50, occupation < =18 yrs indoors & outdoors, 1 mole > =5 mm on right arm, born in NZ, history of NMSC); RR = 17.71 (age > 50, indoor occupation < =18, no moles on right arm, born in NZ, history of NMSC)			
North	0.09	0.37	0.55	0.98	0.07	0.34	1.92	3.23
Midland	0.15	0.43	0.8	1.05	0.11	0.46	2.06	3.24
Central	0.06	0.34	0.59	1.00	0.05	0.31	1.32	2.68
South	0.10	**0.31**	0.51	0.68	0.09	0.36	1.39	2.49
	Low Risk: RR = 1.00 (olive skin, no large moles, don't know if close family with large moles, no personal history of NMSC)				Low Risk: RR = 1.00 (age < = 50, indoor occupation < =18 yrs, no large moles, not born in NZ, no personal history of NMSC); RR = 2.59 (age > 50, indoor occupation < =18 yrs, no large moles, not born in NZ, no personal history of NMSC)			
North	0.01	0.03	0.04	0.08	0.01	0.03	0.28	0.48
Midland	0.01	0.03	0.06	0.08	0.01	0.04	0.30	0.48
Central	<0.001	0.03	0.05	0.08	<0.001	0.03	0.19	0.40
South	0.01	0.02	0.04	0.05	0.01	0.03	0.20	0.37

Calculations for the values in bold are shown in Additional file 2: Appendix 3.

The highest possible combined RR for women was 189.38, corresponding to women who had fair skin, 3 or more moles > =5 mm on their right arm, 1st degree relatives with large or unusual moles and a personal history of NMSC. For the selected ages shown in Table 4, the 5-year absolute risk of developing melanoma for these high-risk women ranged from 0.89% for a 20 year-old living in the Central region of New Zealand, to 14.52% for an 80 year-old living in the Midland region. For the lowest risk women (with none of the melanoma risk factors included in the model), their absolute 5-year risk was very low, even at older ages.

For men aged over 50 years, or for men aged 50 years and under whose occupation was mainly outdoors at age 18 or below, who had 5 large moles on their right arm, who were born in New Zealand and had a personal history of NMSC, the RR of melanoma was 70.31 or 27.19, respectively, compared to men with no risk factors (Table 4). For the selected ages shown, the 5-year absolute risk for these high-risk men ranged from 0.15% for 20 year-olds living in the North, to 12.19% for 80 year-olds living in the Midland region.

For both sexes and all regions of residence, the absolute risk increased with age. The Midland region had the highest absolute risk in most subgroups.

## Discussion

To our knowledge these are the first comprehensive risk assessment models for melanoma developed for a light-skinned population with very high melanoma incidence. The models exhibited good performance characteristics for men and women, suggesting that the models are likely to aid the clinical management of patients concerned about their risk of melanoma. These models included well-recognised risk factors for melanoma (for example, having several large moles) as well as less well-established factors (for example, indoor versus outdoor occupation up to the age of 18 years, in men). The high attributable risk estimates show that the few variables included in the final models captured most of the melanoma risk. The RRs from these models were then included in the calculation of individual 5-year absolute risk of melanoma for New Zealand men and women.

Criteria for defining population groups at high risk of melanoma often rely on lists of risk factors and relative risk estimations frequently seen in aetiological research [18-21]. However, predicting the individual likelihood of an outcome is distinct from explaining disease causality[22]. Prediction models should focus on absolute risk: relative risks are only used as part of the calculation of the absolute probability of the outcome [22]. Moreover, the regression coefficients obtained from a prediction model indicate the mutually adjusted relative contribution of factors to the risk of the outcome [14]. This adjustment is not possible when using only a simple list of risk factors for individual risk assessment.

The design of the case–control study ensured complete reporting of melanomas, and the candidate variables were almost 100% complete. The response rate and cooperation rate in this study were very high for cases (84.5% and 85.5%, respectively) and comparable to other international case–control studies (54.2% and 67.6%, respectively), for controls.

Our results are consistent with what is known about melanoma in New Zealand. However, the prevalence and nature of some risk factors related to sun exposure, for example solaria use, may have changed since 1992–94, so the absolute risks produced from this historical data may not be as applicable now. The risk of melanoma increases with age [23] as do the absolute risks from our models. The higher than expected risk for 20 year-olds in the Southern regions may be related to the high sun exposure of youth in the central, very sunny parts of the region. The three geographical regions that supplied melanoma cases cover latitudes from about 39°S to 42°S, whereas New Zealand covers latitudes from about 35°S to 46°S, but there is no *a priori* reason to expect that melanoma risk factors and their magnitude of risk vary by region. The variability of the underlying risk by latitude was incorporated in the baseline incidence estimates.

Some model overfitting is inevitable with moderately sized datasets, but to compensate for this we used AIC as one measure of model fit. AIC penalises large models and hence reduces overfitting [14]. The two models have been well validated internally. Bootstrap resampling using both forward and backward selection showed that the models were reproducible, and calibration was very good. Internal validation, although essential, does not provide information about performance in other populations: although infrequently done, the models should be externally validated before clinical use.

Few population-based absolute risk predictor models for melanoma have been developed worldwide. Fortes et al. [19] assessed a risk prediction tool in Italian and Brazilian populations, but most other models have not been validated. Fears et al. in 2006 [9] developed an absolute risk predictor for non-Hispanic whites in the US. New Zealand has more than twice the incidence rate of melanoma in the United States and our results show absolute risks several times the comparable risks from the American study [9]. Australia has a melanoma predictor tool available which has been created without the formal development of a multivariable model or identification of the most important predictors so is not comparable to ours [24].

## Conclusions

Melanoma risk in people with multiple risk factors is greater, and sometimes much greater, than in people with no risk factors, and although almost all the risk factors included in the models are unmodifiable, these results, once incorporated into a prediction tool, provide health professionals with a more accurate method of identifying individuals at high risk and thus allow them to offer preventive, surveillance and screening activities appropriately targeted to their absolute risk. These strategies are necessary to help reduce the very high morbidity and mortality from melanoma in New Zealand.

## Competing interests

The authors have no conflicts of interest to disclose.

## Authors’ contributions

MJS carried out the original case–control study and its analysis, conceived and designed the current study, interpreted the results, drafted the manuscript and revised it for intellectual content. CC designed the study, carried out the analysis and revised the paper for intellectual content. BC helped with interpretation of results and revised the paper for intellectual content. All authors read and approved the final manuscript.

## Pre-publication history

The pre-publication history for this paper can be accessed here:

http://www.biomedcentral.com/1471-2407/14/359/prepub

## Supplementary Material

Additional file 1: Appendix 4 Variables assessed in the model fitting process. Click here for file

Additional file 2: Appendices 1, 2 & 3 Attributable Risk Calculations, 5-year Absolute Risk Calculations, Two examples. Click here for file
