# Supplementary material for: Individual risk of cutaneous melanoma in New Zealand: developing a clinical prediction aid
Source: BMC Cancer. 2014 May 22;14:359. doi: 10.1186/1471-2407-14-359 (PMC4038363; doi:10.1186/1471-2407-14-359)
Supplement: Additional file 1: Appendix 4 — Variables assessed in the model fitting process. [file 1471-2407-14-359-S1.docx]

Appendix 4. The variables assessed in the model-fitting process.

1. Region of residence. Bay of Plenty, Hawkes Bay, Nelson-Marlborough

2. Age at diagnosis - continuous

2a. Age at diagnosis – categorical or dichotomized at 50 years

3. Sex

4. Marital status.

5. What is the highest level of education you have reached? school up to 3rd or 4th form, UE Bursary HSC, degree or diploma.

6. Where were you born? NZ, NthEur/UK, Sth Eur, Asia, Africa, Australia, Other.

6a. NZ or not NZ

7. When your skin is exposed to strong sunlight for the first time in summer without any protection how often do you get sunburnt? never, sometimes, usually, always.

8. When your skin is exposed to strong sunlight for the first time in summer without any protection how often do you get tanned? never, sometimes, usually, always.

9. When exposed to sun on your skin (other than face and hands) how often do you use a sunblock to protect yourself from burning? never, sometimes, usually, always.

10. After being exposed to sunlight for a long time over several days without any protection how deep a tan do you get? dark, moderate, mild, only freckled, no suntan.

11. Have you ever been sunburnt enough to get blisters of any sort? Yes, no.

11a. How many blisters of any sort have you had in the last year?

11b. How many in the last 10 years?

11c. How many before the age of 20 years?

11d. Had blistering sunburn in last 10 years? Yes, no

11e. Had blistering sunburn before the age of 20? Yes, no

12. Have there been any particular times or situations in your life where you have been exposed to excessive sunlight over a long time and been repeatedly sunburnt? No, war service, nz immigrant, outside employment, outside leisure activity.

12a. Have there been any particular times or situations in your life where you have been exposed to excessive sunlight over a long time and been repeatedly sunburnt? Yes, no.

13. In the last year have you had any sunburns that have caused pain for more than 2 days (but not blisters)? Yes, no

13a. In the last year how many sunburns have you had that have caused pain for more than 2 days (but not blisters)?

13b. In the last 10 years have you had any sunburns that have caused pain for more than 2 days (but not blisters)? Yes, no

13c. In the last 10 years how many sunburns have you had that have caused pain for more than 2 days (but not blisters)?

14. Up to the age of 18 were your sport or leisure activities mainly indoor, indoor and outdoor, mainly outdoor?

15. Up to the age of 18 were your occupations or other usual daily activities mainly indoor, indoor and outdoor, mainly outdoor?

16. After 18 years old were your sport or leisure activities mainly indoor, indoor and outdoor, mainly outdoor?

17. After 18 years old were your occupations or other usual daily activities mainly indoor, indoor and outdoor, mainly outdoor?

18. What was the natural colour of your hair as a teenager? Black, brown, fair or blond, red or auburn.

18a. What is the natural colour of your hair now? Black, brown, fair or blond, red or auburn, grey.

19. What is the colour of your eyes? brown, hazel, green, grey, blue.

20. What is the colour of your skin in the middle of winter? olive, medium, fair

21. Which diagram is closest to the number of freckles on your face in summer?

22. Number of big moles >5mm on body and legs.

22a. Number big moles. Categorised.

23. How many moles in total on your right arm touch the sides or are bigger than the smaller 2mm circle?

23a. Number small moles on arm. Categorized.

24. How many moles on your right arm are bigger than or touch the sides of the larger 5mm circle?

21a. Big moles on arm. Categorized.

25. Which diagram is closest to how ‘moley’ you are?

26. Phenotype class. Sum of hair, eye and skin colour, and tanning reaction scores. Categorized.

27. Personal history of NMSC? Yes, no

28. Does anyone else in your close family have big (>5mm) or unusual moles? Yes, no, don’t know.

29. Family history of melanoma in 1^st^ degree relative. Yes, no
