# Supplementary material for: Individual risk of cutaneous melanoma in New Zealand: developing a clinical prediction aid
Source: BMC Cancer. 2014 May 22;14:359. doi: 10.1186/1471-2407-14-359 (PMC4038363; doi:10.1186/1471-2407-14-359)
Supplement: Additional file 2: Appendices 1, 2 & 3 — Attributable Risk Calculations, 5-year Absolute Risk Calculations, Two examples. [file 1471-2407-14-359-S2.docx]

**APPENDIX 1: Attributable Risk Calculations**

The equation for calculating the attributable risk for each variable is: [[1](#_ENREF_1)]

where *x*=the total number of cases and *r_i_ =* the relative risk for an individual. When the variable is categorical, the equation can be written

$${AR}_{var}\text{=}1-\frac{1}{x}\sum_{k=1}^{c} \frac{x_{k}}{r_{k}}$$

where *x_k_ =* the number of cases in category *k* and *r_k_* = the relative risk of category *k* (relative to the reference category), of *c* categories. If the variables are statistically independent and there are no interactions,[[2](#_ENREF_2)] the attributable risks for the separate variables can be combined using:

**

where there are *p* variables. The standard error for the attributable risk was obtained through bootstrapping. STATA produces several types of confidence interval from bootstrapping: the usual normal intervals (which assumes the underlying distribution of the population is normal), the percentile distribution (that are better with non-normal situations as they determine the interval limits according to the sample distribution) and bias corrected intervals[[3](#_ENREF_3)]. The percentile confidence intervals were reported in this case since, as there is no evidence of bias in either estimate and, since the attributable risk can only be between 0 and 1, the normal intervals were not practical.

**APPENDIX 2: 5-year Absolute Risk Calculations**

The original formulation is reported by Gail et al[[2](#_ENREF_2)] but the adapted version used here is Fears et al.[[4](#_ENREF_4)] When the calculations use ages that are start points of the 5-year age intervals (20, 25, 30 …) the equation simplifies to

where *r* is the relative risk of melanoma for an individual with particular characteristics, *h*_1i_ is the *i*^th^ age-group-specific baseline hazard of developing melanoma, *h*_2i_ is the *i*^th^ age-group-specific mortality rate, *a* is patient age in years. The original theory splits the ages into 13 intervals: [0, τ_1_), [τ_1,_ τ_2_)… [τ_12,_ τ_13_). So τ_i_=20, 25, 30, etc, for *i*=1 to 13. If *a* = τ_i_ then τ_i+1_=*a*+5.

**APPENDIX 3: Two examples**

Example 1: A 40 year-old woman (age group, *i*=5), living in the South has fair skin, two large moles on her right arm, no close family members with large moles, and no history of NMSC.

From the logistic model, this means the relative risks are:

4.50 for fair skin colour,

1.09 for no close family with large moles,

2.59 for having 2 moles >=5mm on her right arm,

and 1.0 for no personal history of NMSC.

It follows that, as there were no significant interactions, and using more decimal places than are shown here,

*r* = overall estimate of relative risk = 4.50*1.09*2.59*1.0 = 12.66

The relevant baseline incidence rate (from Table 2) is 44.53 per 100,000 population. Therefore,

$$h_{15}=\frac{44.53}{100,000}\left( 1-0.8893 \right)=5\times{10}^{-5}$$

Mortality, *h*_25_, from Table 2, is 101.5 per 100,000.

So, *h*_15_**r* = $6\times{10}^{-4}$

*h*_15_**r* + *h*_25_ = $6\times{10}^{-4}$ + $\frac{101.5}{100,000}$ = $1.642\times{10}^{-3}$

Thus the 5-year absolute risk for this woman is estimated as:

$$P\left( a,r \right)=\left\{ \frac{h_{15}r}{h_{15}r+h_{25}} \right\}[1-\exp\left\{ -\left( \tau_{i+1}-a \right)\left( h_{15}r+h_{25} \right) \right\}]$$

$$=\frac{0.0006}{0.0016}[1-\exp\left\{ -5\left( 0.0016 \right) \right\}]$$

= 0.0031 or 0.31%

Example 2: A 60 year old man (i=9), living in the Midland region worked mainly outdoors when aged <=18 years, has 5 large moles on his right arm, was born in New Zealand, and has a history of NMSC.

From the logistic model, this means the relative risks are:

2.59 for age over 50 years,

1.94 for working mainly outside as a teenager,

(1.15)^5^ for having five large moles on his right arm,

2.21 for being born in New Zealand,

and 3.1 for having a personal history of NMSC.

It follows that, as there were no significant interactions, and using more decimal places than are shown here,

*r* (overall estimate of relative risk) = 2.59***1.94*(1.15)^5^*2.21*3.1 = 70.31

The relevant baseline incidence rate (from Table 2) is 161.28 per 100,000 population. Therefore,

$$h_{19}=\frac{161.28}{100.000} \left( 1-0.8501 \right)=2\times{10}^{-4}$$

Mortality, h_29_, from Table 2, is 1074.51 per 100,000.

So, *h_19_*r* = 0.0002*70.31 = 0.0170

*h*_19_**r* + *h*_29_ = 0.0170 + $\frac{1074.51}{100,000}$= 0.0277

Thus the 5-year absolute risk for this person is estimated to be:

$$P\left( a,r \right)=\frac{0.0170}{0.0277}\left[ 1-\exp\left\{ -5\left( 0.0277 \right) \right\} \right]=0.0794$$

or 7.94%

**References**

1. Bruzzi P, Green S, Byar D, Brinton L, Schairer C: **Estimating the population attributable risk for multiple risk factors using case-control data.** *Am J Epidemiol* 1985, **122**(5):904-914.

2. Gail M, Brinton L, Byar D, Corle D, Green S, Schairer C, Mulvihill J: **Projecting individualized probabilities of developing breast cancer for white females who are being examined annually.** *J Natl Cancer Inst* 1989, **81**:1879-1886.

3. StataCorp: **Stata: Release 11***.* College Station, TX: StataCorp LP: Statistical Software; 2009.

4. Fears T, Guerry D, Pfeiffer R, Sagebiel R, Elder D, Halpern A, Holly E, Hartge P, Tucker M: **Identifying individuals at high risk of melanoma: a practical predictor of absolute risk.** *J Clin Oncol* 2006, **24**(22):3590-3596.
